# Is intensive training with a time interval between instruction and planning CT necessary for deep inspiration breath-hold radiotherapy in breast cancer?

**DOI:** 10.1007/s00066-024-02295-7

**Published:** 2024-09-13

**Authors:** M. Sonnhoff, R.-M. Hermann, K. Aust, A.-C. Knöchelmann, M. Nitsche, B. Ernst, H. Christiansen, R.-M. Blach

**Affiliations:** 1 Department of Radiotherapy, Hannover Medical School, Carl-Neuberg-Straße 1, 30625 Hannover, Germany; 2 Center for Radiotherapy and Radiooncology Bremen and Westerstede, 26655 Westerstede, Germany; 3 Department of Radiation Oncology, University Hospital Schleswig-Holstein, 24105 Kiel, Germany

**Keywords:** Breath guided therapy, Patient training, Cardiac toxicity, Resource management

## Abstract

**Background:**

Breathing instruction and exercises and a time gap between training and planning CT scans (pCT) is recommended as part of deep inspiration breath-hold (DIBH) assisted radiotherapy (RT). However, this is associated with additional time expenditure.

**Materials and methods:**

In two of the authors’ treatment centers (TC), patient training took place before the planning CT of DIBH-assisted therapy. In TC 1, a further appointment was made with a minimum interval of 2 days to perform the planning CT. At TC 2, the planning CT was performed immediately after the first patient instruction. A retrospective evaluation of the clinical parameters of the therapy was carried out to investigate the relevance of the time gap between DIBH exercises and pCT.

**Results:**

A total of 72 patients were included, 35 of whom were treated in TC 1 and 37 in TC 2. In TC 1, an average interval of ~4 days was observed between patient training and planning CT, while in TC 2, training and CT were performed immediately after each other. No significant differences in radiation dose exposure of the lung on the treated side, the whole lung, or the heart were found between the two centers. Furthermore, there was no significant difference in the application of the daily RT fraction. The requirement for daily positioning checks was also the same at both treatment centers.

**Conclusion:**

This study does not show any advantages for a time gap between instruction/training and pCT. Skipping the time break does not deteriorate any clinically relevant endpoints.

## Background

Adjuvant radiotherapy (RT) in breast cancer is an established treatment modality to reduce the risk of cancer recurrence after breast-conserving surgery and to improve oncological outcomes. It is used in the majority of patients [1]. Although recent advances such as partial breast irradiation have helped to enhance patient outcomes and to reduce toxicity, the delineation and protection of organs at risk (OAR), especially the heart and the left anterior descending artery (LAD), still plays an important role [2]. For this purpose, deep inspiration breath-hold (DIBH) is an established method to protect the heart, especially the LAD, in RT of left-sided breast cancer [3, 4]. Patient training as well as a time gap between training and planning CT (pCT) is recommended [5]. However, this often delays the start of therapy, and the training uses additional time and work resources. Furthermore the exact length of this time gap is often center-specific, thus leading to inhomogeneity in the implementation of the process.

The aim of this retrospective study was to analyze whether a break between DIBH training and the start of RT had an effect on OAR protection. For this purpose a retrospective evaluation was carried out between two treatment centers (TC).

## Materials and methods

This retrospective study focused on the analysis of the efficacy and differences of two concepts of DIBH training before adjuvant RT in breast cancer patients.

The standard operating procedures (SOPs) for patient training was specified at each TC under the responsibility of each head of medical physics. Patient training and the relevant SOPs at the different TCs were defined as the following:

### TC 1:

DIBH training and pCT appointment should each be assigned as two separate dates. There should be at least 2 days between the two dates. Both appointments are scheduled in a timeslot of 30 min each. The patient is instructed to perform DIBH exercises independently during the time gap between the training appointment in TC 1 and the pCT.

### TC 2:

Primarily, only one DIBH training date is given here. Once the radiation therapist (RTT) has successfully assessed the training, the pCT takes place immediately after the training. The combined training and pCT appointment are scheduled with a timeslot of least 30 min. The patient is instructed to train independently during the break between the pCT and the start of RT.

The same information material was provided in written form at both TCs.

The requirements for successful training were identical at both locations. The patients had to be able to hold their breath for at least 30 s and to maintain a constant breathing excursion. There was only a time gap between training and pCT at TC 2 in case the breathing was inadequate according to the criteria mentioned above.

The mode of treatment and use of DIBH did not differ between the two TCs. Both facilities shared the same RTTs. RT planning and validation of the plans was carried out in cooperation across both TCs.

In addition, the same linear accelerators (Elekta Ltd, Crawley, United Kingdom) and surface-guided RT (SGRT) systems (Vision RT, London, United Kingdom) were used at both TCs for therapy monitoring of the breathing excursion. Daily cone beam CT (cbCT) was performed up to the 5th treatment day of DIBH-supported therapy. If the patient’s deviation of the positional axes was <3 mm, daily monitoring via cbCT was dispensed with, and further monitoring was done only once a week.

By dose–volume histogram analysis, the mean doses to the entire lung, the ipsilateral lung, and the heart, respectively, were calculated and compared.

In addition, the positional deviation of the isocenter after validation by a cbCT scan in the first, second and last third of the respective treatment series of the patients was recorded.

The time for each TC used for DIBH training and pCT was extrapolated. Furthermore, the time between the patient’s registration at reception until the end of the application of the daily RT (as documented in the authors’ patient management system) was analyzed.

## Statistics

The aim of the study was to examine the non-inferiority of the procedure without a break between DIBH training and pCT.

Descriptive statistics of the treatment parameters were performed. Differences in quantitative parameters were examined using the Wilcoxon test. The odds ratio for the possibility of dispensing with the daily position verification via pCT was calculated. Values were considered as statistically significant at *p* =< 0.05. Statistical analysis was performed using Prism® 10 for Mac, (Version 8.00, GraphPad Software Inc., San Diego, CA, USA.

## Results

The analysis covered the period from August to December 2023 and included all patients at both TCs who received DIBH-supported RT for breast cancer as part of adjuvant therapy. For the evaluation, a total of 35 patients were analyzed at TC 1 and 37 patients at TC 2. At TC 1, two patients needed to be excluded due to a deviation from the center’s SOPs.

At both centers, the majority (29 in TC 1 versus 30 in TC 2) of treatments were carried out in the adjuvant setting after breast-conserving surgery (Table 1).

**Table 1:** Epidemiological data of patients treated at treatment center 1 and 2

	Treatment center 1 (TC 1)	Treatment center 2 (TC 2)
*n*	35	37
Age	61.0 ± 13.91	61.38 ± 12.08
Breast-conserving	29	30
Thoracic wall	5	7
Without lymphatic drainage/with lymphatics	21/13	19/18
Periclavicular	13	17
Mammaria interna region	10	9
Axilla	5	2
Laterality left/right/both sides	31/3/0	27/7/3
Daily cbCT verification yes/no	29 (94.29%) /2 (5.71%)	35 (94.87%) /2 (5.13%)
Hypofractionated/normofractionated	27/7	32/5
Time gap for training [days] (mean ± SD)	4 ± 3	No training break

*cbCT* cone beam CT

The proportion of treatments involving the lymphatic drainage pathways was slightly higher at TC 2 than at TC 1 (TC 1: *n* = 13 vs. TC 2: *n* = 18).

The majority of treatments were carried out as hypofractionated RT with 16 fractions. The seven cases at TC 1 and the five cases at TC 2 that were normofractionated received 28 fractions.

In TC 2, three cases with bilateral breast cancer received RT. These patients were excluded from the evaluation of the mean lung dose (treatment side) and only included in analyses of the total mean lung dose.

At TC 2, no training had to be repeated or paused during the evaluated period. At neither center did therapy need to be postponed due to insufficient patient compliance to DIBH.

In TC 1, the average break between training and pCT was 4 days (SD= ± 3, range 2–15 days). 

**Fig. 1:**
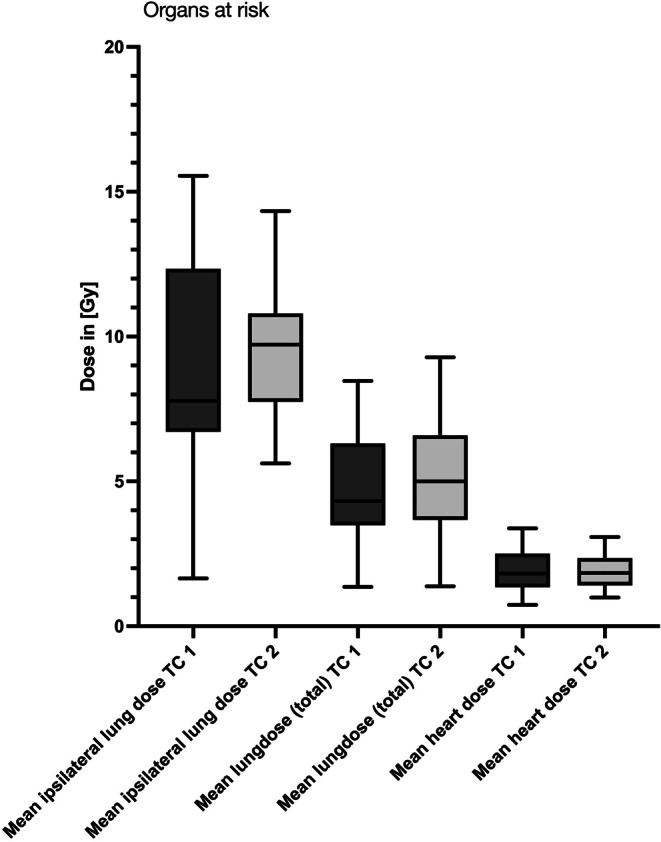
Mean organ doses compared between TC 1 and TC 2. There was no significant difference between the two centers

Evaluating the dose exposure of the OARs, there were no significant differences in the two groups, neither for the ipsilateral mean lung dose (9.095 Gy ± 3.271 vs. 9.593 Gy ± 2.247; *p* = 0.3601) nor for entire lung mean dose (4.906 Gy ± 1.820 vs. 5.150 Gy ± 1.820; *p* = 0.652) nor for the heart mean dose (1.996 Gy ± 0.674 vs. 1.919 Gy ± 0.5901; *p* = 0.0751) (Table 2; Fig. 1).

**Table 2:** OAR exposure and positional deviation after control imaging via cbCT

	Treatment center 1 (TC 1)	Treatment center 2 (TC 2)	T‑test significant?
Mean ipsilateral lung dose ± SD [Gy]*	9.095 ± 3.271	9.593 ± 2.247*	No (*p* = 0.3601)
Mean total lung dose ± SD [Gy]	4.906 ± 1.820	5.150 ± 1.820	No (*p* = 0.4538)
Mean heart dose ± SD [Gy]	1.996 ± 0.6741	1.919 ± 0.5901	No (*p* = 0.0.652)
Anterior/posterior ± SD [cm]	0.2092 ± 1.537	0.2560 ± 0.1926	No (*p* = 0.0751)
Superior/inferior ± SD [cm]	0.2771 ± 0.2235	0.2819 ± 0.2387	No (*p* = 0.9809)
Left/right ± SD [cm]	0.2431 ± 0.2074	0.1741 ± 0.1544	Yes (*p* = 0.0022)
Average duration of daily RT [min] (mean ± SD)	13.31 ± 3.32	13.57 ± 3.91	No (*p* = 0.2101)

*Exclusion of cases with RT of the breast on both sides

**Fig. 2:**
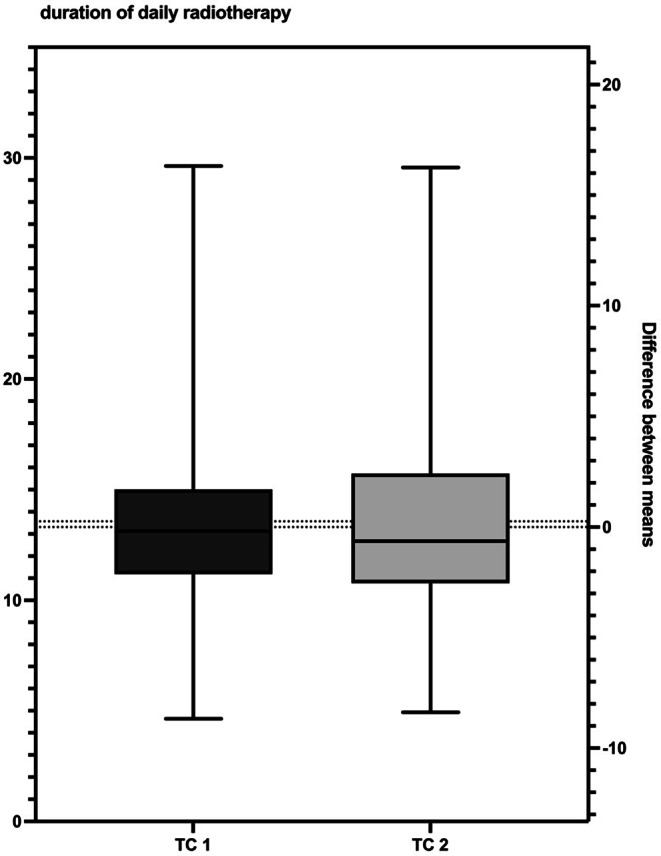
Comparison of the average duration of the daily fraction application. There were no significant differences between the two TCs (*p* = 0.2101)

In two cases, a mean heart dose of 3.38 Gy (TC 1) and 3.04 (TC 2) was accepted in favor of the oncologically necessary therapy (inclusion of the internal mammary chain) in patients with a left-sided primary tumor. The higher than expected heart dose was due to the treatment volume, not due a missing training gap—as this effect was observed at both TCs.

When evaluating the displacement after the control images for the alignment of the isocenter, there was only a significant difference in the left/right axis (*p* = 0.0022). The anterior/posterior axis (*p* = 0.0751) and the superior/inferior axis (*p* = 0.9809) showed no significant differences.

Daily position verification via cbCT was required in 94.29% of TC 1 and 94.87% of TC 2. The odds ratio was 1.125 and the difference between the TC 1 and TC 2 groups was not significant (C95% 0.1239 to 6.375, *p* = 0.9120). The average time of radiation application was 13.31(± 3.32) min at TC 1 and 13.57 (± 3.91) min at TC 2 (Fig. 2). There were no significant difference in the duration of daily RT application between the two TCs.

## Discussion

A total of 72 patients were included in the analysis. In 35 patients, there was a break between the DIBH training appointment and the pCT. In total, 37 patients received the pCT procedure immediately after DIBH training. It was possible to implement DIBH-supported therapy in all patients. In TC 1, this was associated with a scheduled working time of about 35 h; in TC 2, training and pCT could be carried out in 18.5 planned hours.

No significant differences were found between the two facilities with regard to OAR doses. With regards to displacement, only a significant difference in the average displacement in the r/l axis was recorded in the evaluation. Furthermore, no difference was seen between the duration of the daily radiation fractions.

The reduction in mean cardiac dose with DIBH-supported RT compared to free breathing is evident [6]. The risk of cardiac mortality is linearly dose-dependent [7]. A mean cardiac dose of less than 3 Gy is considered safe to decrease the risk for cardiac toxicity at an acceptable minimal level [8].

With two exceptions, an average cardiac dose of less than 3 Gy was achieved in all patients. In both cases, there was an indication for RT of the left breast including the supra- and infraclavicular lymphatic drainage pathways as well as the internal mammary chain. Adequate dose coverage could not have been achieved without exceeding the 3 Gy constraints of the heart.

In the lung, the dose recommendations and dose limits could be maintained, which is in line with the QUANTEC data [9]. Overall, the therapy was carried out in both centers in compliance with the clinical guidelines and recommendations, regardless of time gaps between DIBH training and pCT.

Evaluating the positioning data, it was not possible to identify any significant differences in the A/P and S/I axes in both groups. Especially in the error-prone A/P axis, there was no relevant difference between TC 1 and TC 2 [10].

The L/R axis showed a significant difference between the two groups. However, no plausible cause related to a time gap could be found. Furthermore, intraobserver variability in daily positioning was ruled out, as the personnel was deployed at both TCs and changed between the locations on a weekly basis. The alignment in the L/R axis only plays a minor role in the daily initial positioning on the couch and is also directly compensated by the positioning correction after the cbCT. The clinical target volume (CTV) to planned target volume (PTV) margin used at the authors’ centers are in line with the recommendations found in the literature [11]. They are sufficient to compensate for positioning uncertainties despite cbCT and SGRT. Finally, the differences in all axes were within the tolerance for SGRT as described in the literature [12].

In addition to adjusting the positioning axes, the cbCT is also relevant for validating the relationship between target volume and OAR [12]. The daily cbCT for the first RT fractions is used for online set-up correction to implement a stable and reproducible DIBH application of the daily RT fraction [13]. According to the authors’ internal guidelines, only two patients per TC qualified to reset a daily position control and received a weekly cbCT control. There was no significant difference between the two TC patients and no benefit from a break between training and pCT. In addition, a daily positioning control via cbCT is associated with additional time required for the application of the daily fraction dose. However, according to the current evaluation, a time gap between DBIH training and pCT neither reduced the need for positioning checks via cbCT nor saved worktime. The focus is on the safety of the RT fraction and the indication for cbCT within the limits of application, which makes the potential for saving worktime very low.

The use of RT to reduce a local recurrence must be weighed against the risk of the therapy causing a secondary malignancy [14].

From a radiation protection point of view, cbCT frequency must also be discussed as a relevant dose exposure [15].

As part of the internal quality measurements, the authors specified a computed tomography dose index of 0.3 mGy. The increase in the risk of a second malignancy due to a daily positioning correction via cbCT control, which can be found in the literature [16], must be weighed against safe interfractional application [13]. However, the technical reduction of low-dose exposure of the normal tissue could be a more efficient way to reduce the risk of secondary malignancies than omitting the cbCT [17].

The implementation of DIBH training is recommended and has been shown by Kalet at al. [5]. However, the present evaluation assumes that a time gap between DIBH training and pCT offers no further advantage. There is also no evidence that patient compliance increases due to a timegap between the training and the pCT. The time required for the application of the daily fractional dose is the same at both centers. Additionally, no DIBH-supported therapies had to be postponed at either center during the observed period. Finally, the OAR exposure also reflects an adequate implementation of DIBH at both centers. Thus, the authors do not observe any disadvantage in patient compliance due to the omission of the timegap between DIBH training and the pCT.

A significant benefit from a time gap between DIBH training and pCT in daily RT application did not show any benefits in the current TCs: neither qualitatively in view of OAR exposure and positioning accuracies nor quantitatively in respect to time-saving. Furthermore, in terms of resource management, the planned time required for treatment planning process was halved by omission of the time gap. From the authors’ point of view, the most effective action to save resources is to omit the additional DIBH training session and to combine the training directly with the pCT.

This study has limitations, as it is a retrospective comparison of the clinical routine at the authors’ TCs, and not a prospective study protocol. This is particularly noticeable in the high dispersion of the time intervals between training and pCT.

However, real-world data from routine clinical practice were used as part of the evaluation and thus it was possible to show that omitting the break between training and pCT does not result in any disadvantage for the patient with regard to the relevant endpoints of the evaluation.

In conclusion, the authors consider it practicable to combine DIBH instruction/training and pCT in one appointment with the advantage of saving time in terms of the amount of work involved without any clinically disadvantage for the patient.

## Abbreviations

CbCT: Conebeam CT
DIBH: Deep inspiration breath-hold
LAD: Left anterior descending artery
OAR: Organ at risk
pCT: Planning CT
RT: Radiotherapy
RTT: Radiation therapist
SGRT: Surface-guided radiotherapy
TC: Treatment center
